# Application of SDN Network Traffic Prediction Based on Speech Recognition in Educational Information Optimization Platform

**DOI:** 10.1155/2022/5716698

**Published:** 2022-07-22

**Authors:** Susheng Zheng, Shengxue Yang

**Affiliations:** Jiangxi University of Technology, Nanchang 330098, China

## Abstract

This paper constructs a SDN network traffic prediction model based on speech recognition and applies it to the educational information optimization platform. By analyzing the influencing factors of SDN network equipment, communication links, and network traffic, this paper constructs the initial index set of SDN network traffic situation. In the data plane of SDN, the queue management algorithm is used to control the flow. On this basis, an IRS mechanism is proposed based on the advantages of SDN centralized control and the difference of transmission performance requirements between large and small streams. For the transmission of large traffic, IRS adopts greedy routing and multipath routing based on the remaining bandwidth to make the traffic evenly distributed in the network, and IRS adds the scheduling strategy based on IP addressing to avoid packet disorder. Simulation results show that the effectiveness of this algorithm can reach 95.67% at the highest, and the MSE convergence is 0.0021 at the lowest. At the same time, this method completes the quantitative evaluation of SDN network traffic situation, effectively solves the problem that SDN traffic situation labels cannot be determined, and opens a new vision of global state observation for SDN network management. This research can provide some technical support for the educational information optimization platform.

## 1. Introduction

In the knowledge economy era, education directly transforms into the mechanism for resource formation and allocation as well as the capacity to possess and distribute capital. An unstoppable trend of networked development of global information resources has been brought about by the quick development of information technology [1]. The main resource in modern society is information. It will be challenging to maximize economic and social benefits if resource development and use are not brought into the economic operating system. With the assistance of the development of deep NN (neural network) technology [2–4] in the new century, speech recognition technology has achieved ground-breaking successes. Speech recognition's primary function is to translate the data present in a speech signal into the corresponding text data. The ultimate objective is not to translate speech into words. Making machines understand humans is the aim of speech recognition. Future research may focus more on how to integrate semantic understanding with speech recognition. The four main components of the speech recognition framework are the decoder, language model, acoustic model, and feature extraction and processing module of the speech signal. Information technology professionals are currently considering how to better apply speech recognition technology to the field of education and teaching due to the technology's rapid development. The phonetic teaching approach is straightforward and effective in the educational setting. As a result, research into the use of phonetic recognition in the educational setting has gained importance and drawn a lot of attention from scientists. Economic and social benefits are very likely to result from it. With the general advancement of artificial intelligence, speech recognition technology will inevitably become more sophisticated. To advance the deep integration of information technology and education and teaching, speech recognition technology will be extensively applied to all facets of teaching and learning in the future. Studying the use of SDN network traffic prediction based on speech recognition in the educational information optimization platform is therefore important from both a theoretical and practical standpoint.

The importance of traffic control engineering as a mechanism for enhancing network performance has grown as a result of the diversified development of network applications, the expansion of services available over networks, and the constant rise in bandwidth demand. In addition, the unit integration mechanism used by traditional network management is unable to accurately describe the network traffic situation, and network resources have subpar global performance capabilities [5]. The question of how to increase network throughput, decrease network delay, and implement flexible scheduling to solve the traffic optimization problem of the data center network has become urgent due to the increase in traffic volume, the number of application deployments, and the increased requirements for service quality. OpenFlow, an SDN technology that has recently emerged, has created a new opportunity to address this issue [6]. A new network architecture called SDN divides network control and forwarding tasks. It can make network administration easier and increase the network's programmability and flexibility. SDN networks are continuously being developed on the foundation of traditional networks, and they have the potential to significantly increase network resource utilization [7]. SDN is a concept rather than a specific technology that refers to designing network architecture with the separation of forwarding and control. It plans the relationships and interfaces between each level of the network's hierarchy. Uneven traffic distribution in the data center network is one of the factors contributing to network congestion, which can be minimized by using a routing strategy that distributes traffic evenly among several accessible paths [8]. Currently, most multipath routing strategies use static hashing or shunting to divide data streams among different paths, which is easily problematic and can result in issues like large stream conflicts or packet disorder. This paper suggests a speech recognition-based SDN network traffic prediction model based on this and applies it to the platform for optimizing educational information. The innovation of this paper is as follows:

1. This paper summarizes the application of speech recognition technology in the optimization of educational information resources. By analyzing the influencing factors of SDN network equipment, communication links, and network traffic, the initial index set of SDN network traffic situation is constructed. In the data plane of SDN, this paper uses queue management algorithm to perform flow control. The same set of parameters is used to implement the same management strategy for different data stream packets, and the influence of packet arrival and departure in buffer area on queue length is also considered. Experiments show that the network parameters calculated according to this model can meet all kinds of network requirements.
2. In this paper, the data acquisition module in the SDN controller is designed, and the SDN network platform is built, which realizes the data acquisition and storage of traffic situation indicators. Aiming at the problems of packet disorder, this paper proposes an IRS mechanism based on the advantages of SDN centralized control and the characteristics of different performance requirements of large and small streams. For large streams, IRS uses greedy routing and multipath routing based on remaining bandwidth to make the traffic evenly distributed in the network, and IRS adds a scheduling strategy based on IP addressing to avoid packet disorder.

This paper will be broken up into five sections in accordance with the needs of the research. Each section contains the following primary work and organizational structure. Introduction is the first section. This section explains the background of the research, the research's subject matter, its originality, and its organizational design. The second section provides an overview of the state of research both domestically and internationally before moving on to discuss the research's main points and ideas. The third section is divided into two sections. The optimization of educational information and network traffic forecast are related topics that are covered in Section 3.1. Using speech recognition, Section 3.2 creates a model for SDN network traffic prediction. The model that was built in this paper is repeatedly simulated in Section 4, and the outcomes are examined. Summary and prospects are covered in the fifth section. It mainly elaborates on the research findings from this paper and anticipates the challenges ahead as well as the work that will come after.

## 2. Related Work

Shu et al. explored the working method of speech recognition technology and its teaching application, and emphatically introduced several main forms of speech recognition technology in the field of education and teaching. The current problems of speech recognition technology and the feasible way for future development to mature are discussed [9]. Ji et al. combined rough set analysis, DL (deep learning), and other methods to evaluate and predict the SDN network traffic situation [10]. The experimental results show that the model has high prediction accuracy. Chan et al. proposed an optimization algorithm based on color grouping for the data layer [11]. The model classifies different kinds of data streams, marked with different colors. Jha et al. proposed a new SDN network traffic situation assessment method [12]. This method uses the AK-means clustering method to determine the decision-making attributes of the traffic situation factor matrix and constructs a rough set analysis information system for situation information reduction and attributes importance to calculation and finally generates an unsupervised traffic situation assessment model. Konstanteli et al. conducted an in-depth analysis of network traffic control methods and proposed an algorithm-based traffic control model [13]. It points out the deficiencies in the flow control of the network from the control layer and the data layer. Liu et al. designed a multitenant-oriented network resource allocation and management platform based on SDN [14]. The platform provides its own view interface for different tenant networks and implements tenant resource allocation and traffic isolation. Based on the SDN network structure, Fabrizio et al. proposed the main functional modules of the SDN network traffic situation assessment and prediction system, including the data collection module and the situation assessment and prediction system module, and introduced the functions and implementation methods of each module in detail [15]. Chun et al. proposed a new model of SDN traffic control, which is based on the multicommodity flow algorithm and queue management algorithm to control SDN network traffic more finely [16]. Qi et al. proposed a maximum entropy network traffic prediction and controller predeployment model based on hidden Markov optimization [17]. The model classifies SDN traffic according to the type of protocol, uses the captured historical data flow, uses the maximum entropy algorithm to predict the distribution of future data flow, generates the predeployment scheme of various controllers in the control plane, and joins the hidden Markov chain which optimizes the timeliness of the forecast plan. Compared with the SVR model, this model has higher prediction accuracy, and the generated predeployment scheme can adapt to the dynamic changes in the complex SDN environment.

This paper builds an SDN network traffic prediction model based on speech recognition and applies it to the educational information optimization platform using research from related literature. This paper builds the initial index set of SDN network traffic situation by examining the influencing factors of SDN network hardware, communication links, and network traffic. This paper suggests an IRS mechanism based on the benefits of SDN centralized control and the characteristics of various performance requirements of large and small streams in order to address the issues of packet disorder. In order to evenly distribute traffic across the network for large streams, IRS employs greedy routing and multipath routing based on available bandwidth. Additionally, IRS incorporates a scheduling strategy based on IP addressing to prevent packet disorder. In this paper, flow control is implemented in the SDN data plane using a queue management algorithm. Studies reveal that the network parameters computed using this model can satisfy a variety of network needs.

## 3. Methodology

### 3.1. Application of Speech Recognition Technology in Optimization of Educational Information Resources

The ultimate goal of the application of modern information technology in education is to improve the educational efficiency. The utilization rate of educational resources has been improved, and the number of people enjoying educational information resources has increased geometrically. Education is essentially the process of information transmission [18]. The extensive, continuous, and in-depth influence of modern information technology on education has created a brand-new educational form-information education. At present, in the field of information research, the optimal allocation and efficient development of educational information resources is one of the frontiers and hotspots, and it is of great practical significance to implement it in the subject practice of educational scientific research. Educational information resources under modern information technology refer to the collection of useful information that is selected, organized, and ordered by human beings and is suitable for learners to effectively develop among themselves. With modern information technology, educational resources all over the world can be connected into an information ocean for the majority of educational users to share [19]. The educational information environment includes four elements: people, educational information resources, educational information technology, and educational information norms. These four elements and their many factors constitute a sustainable and dynamic educational information environment system. The effective use of educational information can help students to effectively reconstruct their knowledge structure, strengthen their self-learning and self-management, and at the same time help teachers and administrators to conduct in-depth research in teaching management. The content of modern information resources is very rich, among which network education resources are the most important part of modern education information resources. It has many types, including educational websites, e-books, electronic journals, virtual libraries, virtual software libraries, news groups, and electronic encyclopedias. Computer network technology is increasingly infiltrating into people's daily life, and digital survival has become a brand-new way of life. While people enjoy the surprises brought by the information society, they also expose their poor adaptability to the information society. This is manifested in students and teachers who generally lack the knowledge and experience of information retrieval, identification and integration, and the concept of online security is also very indifferent, and they do not know how to consciously abide by the norms of online behavior. Speech recognition technology is a technology that converts speech signals into corresponding commands so that computers can understand them. With the rapid development of speech recognition technology, the application scenarios of speech recognition have become more extensive, and it has different tasks and functions in various fields, resulting in different design schemes for different application scenarios, but the speech recognition systems all have the same overall structure.

**Figure 1:**
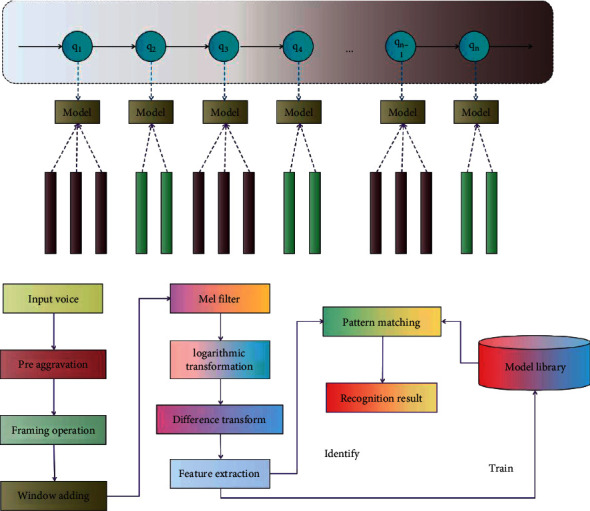
Framework and process of the speech recognition system.

The process of simulating human information exchange by using a computer mainly goes through the following processes: (1) natural language generation, (2) speech synthesis, (3) speech recognition, and (4) natural language understanding. The main task of speech recognition is to identify the information contained in the speech signal into the corresponding text information. The three main components of speech recognition are feature extraction, pattern matching, and reference pattern library. Although phonetics has been around for a while, it has not been widely used because of the difficulty of the corresponding model base and phonetic knowledge. The process of fairly classifying, organizing, managing, and processing the gathered complex information is known as information architecture. Its primary responsibility is to combine the components of information expression, determine its course, and edit the expression's content in order to create a cognitive link between users and the data. Academic and industrial researchers are enthusiastic about DL theory due to its superior modeling capability for complex data. The performance of models based on DL has also significantly improved in many real-world application fields, particularly in speech recognition [20]. The computer will automatically produce the recognition results following preprocessing, feature extraction, training, pattern matching, and other units. From this procedure, it is clear that speech recognition is essentially a pattern recognition system and that the effectiveness and precision of recognition depend on the quality of the speech template and algorithm. Network media's ability to disseminate information quickly and with a wide range of influence is improved by the network's rapid growth and update frequency. Because educational information resources are highly time-sensitive, it is important to gather, process, and use educational information as soon as possible. The framework and process of speech recognition system are shown in Figure 1.

Although teaching itself is a systematic process with a clear goal, producers and consumers of educational information frequently do not interact directly, and many producers frequently produce educational information in accordance with their own intentions. Speech recognition technology has undergone significant technological changes recently due to the growth of the DL field, and the acoustic model has gradually shifted from the conventional Gaussian mixture model to the NN model [21]. The NN model significantly boosts the speech recognition system's recognition performance, enhancing its ability to support human daily life and industrial production. People are accustomed to understanding information in the context of their interpersonal relationships, and they selectively gather, arrange, and assess the information's relevance in light of their own needs. The degree to which information resources are accepted by learners and how well they are accepted by them largely depend on how well the information resources correspond to the learners' own concepts and knowledge bases. The speech recognition industry currently uses a lot of NN-based speech systems. Neurons, the training algorithm, and the network structure make up the majority of NN. Speech recognition suffers greatly from noise and interference, which lowers the rate of recognition. Due to the issues of comprehensive teaching content, rich and diverse speech expressions, and relatively small speech data sets in the educational scene, applying speech recognition to the educational scene is still a difficult research project. The high recognition rate is currently stuck at the “near-field speech recognition” stage and is unable to advance to the stage of “far-field speech recognition,” which is the mode of natural man-machine interaction. The proliferation of inaccurate information and the challenge of finding high-quality resources are issues that we must simultaneously address despite the fact that education technology expands the learning environment for students. The environment for educational information needs to be optimized. A complete speech recognition system usually consists of three parts: (1) the speech front-end processing part responsible for processing speech signals, (2) the language model and acoustic model, which are trained by text corpus and voice data set, and (3) speech recognition, that is, pattern matching.

### 3.2. Construction of the SDN Network Traffic Prediction Model Based on Speech Recognition

**Figure 2:**
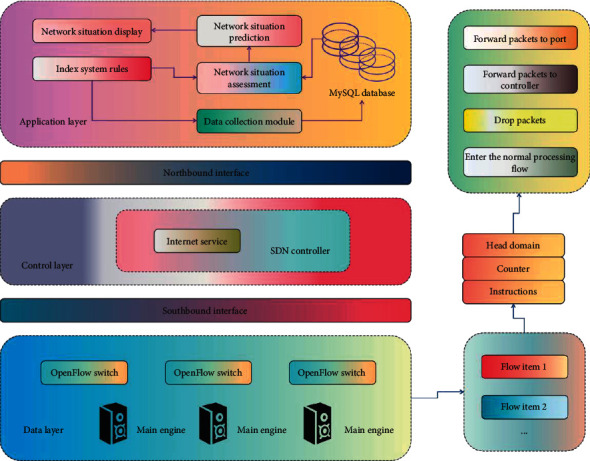
SDN network structure.

Traditional network has been restricted in function expansion and network configuration. In order to make network management more convenient, SDN network has begun to appear in public view. SDN is a new network architecture that separates network control and forwarding functions, which can simplify network management and improve network programmability and flexibility. SDN can maximize the utilization of network resources, restrain the unlimited expansion of network infrastructure, and shield the complexity of the underlying network for the upper users. It simplifies the configuration and management of the network and promotes the innovation and development of the network. SDN network is continuously developed on the basis of traditional network, and the utilization rate of network resources can be greatly improved through SDN network. The latest development of SDN network and switching technology can make the network protocol stack abstract from the physical topology level to the flow level for business control. SDN carries the idea of designing network architecture for forwarding and control, rather than a specific technology. It plans the hierarchical structure of the network and the relationships and interfaces among all levels. With the popularization and development of this concept, different participants have further enriched its content, and they have different understandings of it from different angles. Network traffic will change with time and space. In the time dimension, the network traffic will change with different time periods of the day, for example, the traffic in the daytime is higher than that in the night. In addition, the network traffic may change dramatically in a very short time. SDN network is characterized by hierarchical design of data forwarding and network control. The SDN controller is interconnected with each switch, and control information is uploaded and sent through OpenFlow protocol. SDN network is divided into three layers: application layer, control layer, and data layer. It includes commercial applications used by users in the application layer through which network services can be provided to end users. The control layer consists of one or a group of controllers and connects the data forwarding layer with the application layer through an interface. The control layer has mastered the core, can obtain the global topology of the whole network, and design strategies to control the transmission path of the data layer. The data forwarding layer is composed of switches, hosts, and other underlying network devices, which is the realization of the data plane. The data layer does not have the function of selecting the forwarding path but can only transmit information according to the forwarding path provided by the control layer. The network structure of SDN is shown in Figure 2.

SDN network breaks through the limitation that the service interfaces of traditional network hardware devices cannot be configured and adjusted uniformly and in real time. Its advantages are mainly reflected in two aspects: (1)SDN separates the control plane and the data plane of the current network; the data plane is simply forwarded by the actual network equipment, and the control plane is controlled by a logically centralized controller. This simplifies the implementation and optimization of the strategy and the configuration of the network. (3) SDN improves the programmability of the network system by providing developers with powerful and extensible programming interfaces. Network flow is a dynamic variable that is constantly changing from a fine-grained perspective, but it is comparatively stable over time. If the load balancing model only takes into account this change based on the current time point, without taking into account the change trend of the future data flow, it may result in low cost performance controller migration. However, this migration cannot guarantee the overall improvement of the network performance in a specific amount of time, as it only supports the current burst network data flow, which may increase the overall network delay. Additionally, the load flow may exceed the network capacity when the SDN network uses the optimized routing configuration, particularly when the network experiences burst flow. In order to prevent this kind of congestion, which is the main goal of traffic control, we must allocate suitable transmission resources for data traffic in accordance with its transmission requirements and restrict some traffic flows from entering the network at the bottleneck links. Hedera, a dynamic traffic scheduling strategy that can sense the traffic size, is a representative scheme for addressing the issue of network congestion brought on by heavy traffic collision in an SDN-based data center network. Hedera's strategy is to use centralized scheduling for large flows with long durations and small quantities and default ECMP routing for small flows. To determine the flow rate and size of the large flow in this routing scheme, the edge switch using OpenFlow technology first counts the data of the flow generated by the source.

From the perspective of communication theory, obtaining the best decoded word sequence in speech recognition needs to be transformed into a pattern recognition problem. The core idea of solving the word sequence with the highest probability is to adopt the maximum posterior probability discrimination, and its calculation formula is as follows:

(1) $$\begin{array}{c} \hat{W}=\underset{W}{\text{argmax}}P\left(W|X\right). \end{array}$$

In the speech recognition problem, *P*(*W|X*) is difficult to calculate directly, and it can be converted into the following formula by using the Bayesian formula:

(2) $$\begin{array}{c} \hat{W}=\underset{W}{\text{argmax}}\frac{P\left(X|W\right)P\left(W\right)}{P\left(X\right)}\\ =\underset{W}{\text{argmax}}P\left(X|W\right)P\left(W\right), \end{array}$$

where *P*(*X*) represents the prior probability of the feature vector, which is a constant for all word sequences, so it can be discarded. *P*(*X|W*) represents the probability of using a given word sequence to generate the corresponding acoustic feature vector, which is modeled and calculated by the acoustic model. *P*(*W*) is the prior probability of the word sequence, which can be modeled and calculated by the language model. After framing the speech signal, it is necessary to continue windowing the speech signal to deal with the nonstationary characteristics of the speech signal. The most commonly used window adding methods are the rectangular window and Hamming window. The definition of rectangular window function is shown in the following formula:

(3) $$\begin{array}{c} w\left(x\right)=\left\{\begin{array}{c} 1,\;0\leq n\leq N-1,\\ 0\;\text{other}. \end{array}\right. \end{array}$$

Define the currently available bandwidth *c*(*p*) for path *p*:

(4) $$\begin{array}{c} c\left(p\right)=\underset{e\in p}{\text{min}}c\left(e\right). \end{array}$$

In the formula, *c*(*e*)=*x*(*e*) − *u*(*e*), which is the remaining bandwidth of link *e*. Define the vulnerability of path *p* as *α*(*p*):

(5) $$\begin{array}{c} \alpha \left(p\right)=\underset{e\in p}{\text{max}}\alpha \left(e\right). \end{array}$$

In the formula, *α*(*e*) is the number of paths in different *p*_1_*s* passed on the link *e*:

(6) $$\begin{array}{c} \alpha \left(e\right)=\sum_{l=1}^{L}\delta_{l}\left(e\right). \end{array}$$

Among them,

(7) $$\begin{array}{c} \delta_{1}\left(e\right)=\left\{\begin{array}{c} 1,\;\text{if}p\in p_{1}^{k},\;e\in p,\\ 0,\;\text{else}. \end{array}\right. \end{array}$$

Network bandwidth utilization refers to the average of the ratio of link load to link bandwidth of all links in the recent period. The calculation formula of network bandwidth utilization is

(8) $$\begin{array}{c} \text{Link}\_\text{utirate}\left(t\right)=\frac{\sum_{l=1}^{L}\left(\text{portBytes}\_Rx_{l}\left(t\right)*100\%/{\text{bandwidth}}_{l}\right)}{L},\\ L=1,2,3,\ldots ,L. \end{array}$$

Among them, bandwidth_*l*_ refers to the bandwidth of the *l*th link, that is, the maximum number of bits that can theoretically be transmitted by the physical channel per unit time. portBytes_*Rx*_*l*_ represents the number of bits transmitted by the sender of the *l*th link. The link transmission delay refers to the maximum value of the communication delay from the sending port to the receiving port of all links. The calculation formula is

(9) $$\begin{array}{c} \text{Link}\_\text{delay}={\text{max}}_{i}\left\{p\text{topDelay}\left(i\right)\right\}. \end{array}$$

Among them, the network delay can be calculated by the maximum transmission delay of the link, that is, the maximum time interval *p*topDelay=*T*_2_ − *T*_1_ between the time *T*_1_ of the transmission data packet leaving the source point and the time *T*_2_ arriving at the destination in the network; *p*topDelay represents the end-to-end delay. Network throughput refers to the total number of bytes passing through the switch port connected to the host in unit time without packet loss. It can be calculated by the total number of bytes imported and exported, namely,

(10) $$\begin{array}{c} \text{Net}\_\text{throughput}=\frac{\left(\text{dataBytes}\_\text{inNum}+\text{dataBytes}\_\text{outNum}\right)}{T}. \end{array}$$

Among them, *T* represents the sampling interval; dataBytes_inNum represents the number of incoming bytes connected to the host; and dataBytes_outNum represents the number of exported bytes connected to the host.

In this paper, the machine learning algorithm is used to compare the actual data stream with the predicted data stream, and if the deviation is greater than the set threshold, the deployment scheme is regenerated. Otherwise, the old scheme is still adopted. During the running of the scheme, the controller does not migrate any more to reduce the impact of migration on network performance. In an SDN network, all network resources are pooled together, and the controller distributes path and bandwidth resources evenly among the network's data streams. The data streams processed by the switch will be stored in the buffer area for transmission, and its only responsibility is to forward the data streams in accordance with the controller's defined policies. The network topology discovery module's network topology information and the network monitoring module's link remaining bandwidth information are used by the large flow routing calculation module in the SDN controller to determine the convergence layer link pair of the large flow's source and destination using the greedy algorithm. In this paper, the situation is assessed using a model based on rough sets and clustering [22], which can improve decision tables and assess the current network traffic situation in accordance with the decision rules derived from rough set analysis theory. The historical situation value and current situation value in the SDN network operation process are finally obtained by investigating the calculation method of situation index weight based on attribute importance, allowing for an intuitive and unmistakable understanding of the global network view and network operation trend through the situation value.

## 4. Result Analysis and Discussion

Through Hurst index calculation of the SDN network traffic situation value, the sequence H value is 0.88, which indicates that the situation value sequence has strong self-similarity and long correlation. In order to verify the effectiveness of SDN network traffic prediction model based on speech recognition, this paper designs several simulation experiments on the MATLAB simulation platform. The platform can automatically configure network controllers, switches, etc., and execute protocols. In the simulation experiment, the controller with visual boundary is installed as the control layer of the network. Based on Python programming language, network switches and terminals can be easily configured on Floodlight. At the same time, in this chapter, a large number of comparative experiments are made between the prediction model in this paper and other different prediction models including the convergence of training iteration of traffic situation, the relative error of situation value prediction, the influence of super parameters of the model, and the generalization ability of different data sources. The simulation parameters are shown in Table 1.

**Figure 3:**
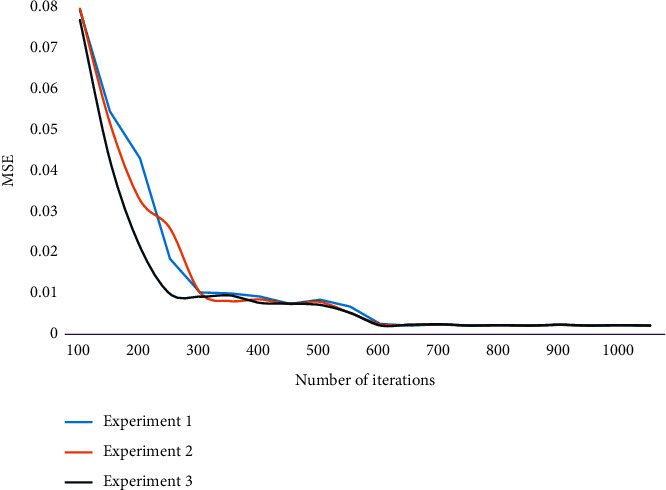
Changes of training MSE in the iterative process of network training.

When a large number of data flows pass through SDN network, network congestion will occur. On this basis, this chapter simulates and analyzes the pet control algorithm based on queue management. Because the network flow is complex and changeable, and many artificial subjective predictions are difficult to be verified, constructing the probability matrix through objective historical data to obtain the probability matrix under the condition of maximum entropy as the prediction result at that time can maximize the accuracy of the prediction. During the experiment, the training sample group is input to train the prediction model, and the maximum number of iterations is set to 2000. The change of training MSE (mean square error) in the network training iterative process is shown in Figure 3.

It can be seen that the MSE is decreasing from the initial 0.08 or so, and it reaches convergence after 600 iterations, and the MSE at convergence is 0.0021. This shows that with the increase of iteration times, the weight update can converge in the process of gradient descent, and the convergence speed is faster.

**Figure 4:**
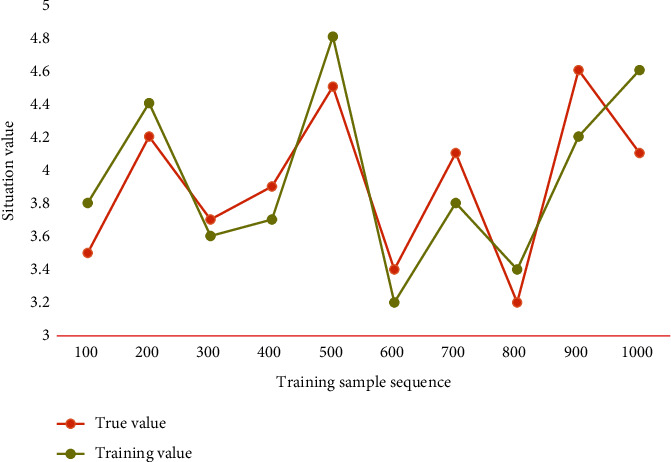
Effect of network training.

First, the parameters are compared and analyzed. Then the maximum and minimum threshold values are changed, and the performance parameters of each packet are further analyzed. Under the condition that the buffer size is constant, this chapter changes the minimum threshold of queue length. The network training effect is shown in Figure 4.

**Figure 5:**
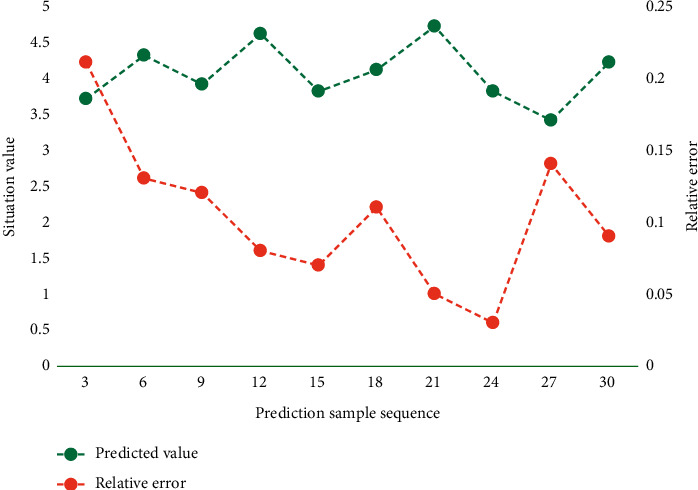
Prediction effect of the model.

For this paper, the main factors that affect traffic prediction are the number, time, and protocol type of packets. Therefore, the model takes packet size, packet protocol name, and time as eigenvalues and generates the network flow data matrix by sorting the data. The trained network model is used to predict 20 groups of untrained samples. The prediction effect is shown in Figure 5.

It can be seen from Figure 5 that the prediction model can achieve good prediction results for any group of traffic situation samples to be predicted, and the relative error of prediction is basically controlled within 15%. In this paper, network throughput, average flow completion time, the remaining bandwidth jitter in Pod and remaining bandwidth jitter in the core layer are selected as performance evaluation indexes. Among them, the average stream completion time refers to the average time taken to complete all 100 MB data block transmissions, and the smaller the time, the better the performance. Network throughput refers to the amount of data transmitted and successfully accepted through the network per unit time in the network. The larger the data, the better the performance. The jitter of the remaining bandwidth in the Pod and the jitter of the remaining bandwidth in the core layer refer to the standard deviation of the remaining bandwidth of each link in the Pod and the core layer, respectively. The smaller the jitter, the better the equalization performance.  Table 2 shows the test results of each index.

**Figure 6:**
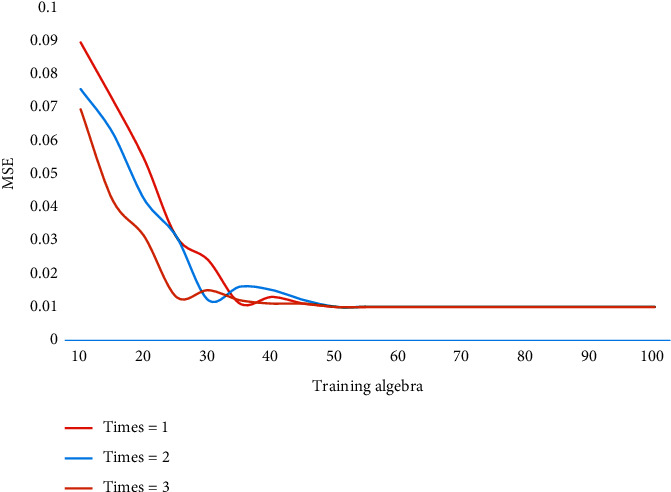
Comparison of the iterative convergence effect.

On the whole, the traffic balancing effect of IRS is better than that of ECMP, which is mainly because IRS uses the dynamic routing method based on the remaining link bandwidth in convergence layer of the network, and large flows are always routed to low-load paths. Moreover, IRS uses the shunting method to route large flows in the core layer, which further balances the load in the network. Because the model uses random initial weights to initialize the network, different initial weights may have different effects on the network iterative convergence. In this paper, the results of three experiments are compared. Times means times, and the values are 1, 2, and 3. The iterative convergence effect is shown in Figure 6.

**Figure 7:**
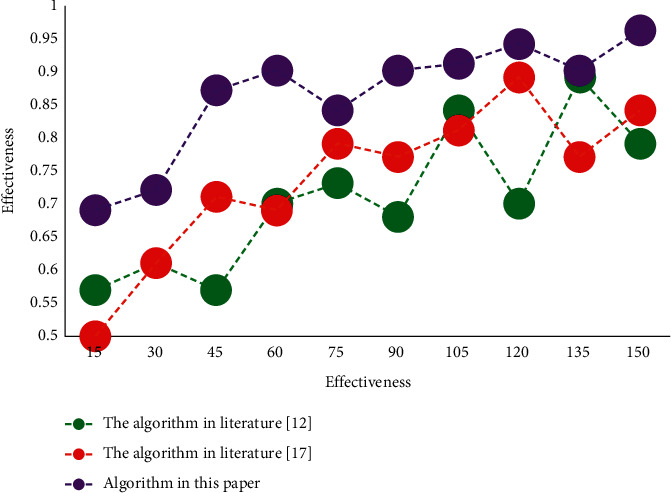
Effectiveness test results of the algorithm.

From the comparison results of curve slopes, it can be seen that the convergence speed of this model is faster than that of the other two models in the three experiments. At the same time, the initial MSE of this model is obviously better than the other two models. It shows that the algorithm in this paper can effectively improve the convergence speed of network model, and the training effect is better. The SDN controller only selects the forwarding path for the new flow injected into the network according to the usage of the remaining flow table of the switches on the feasible path in the current tenant network. The effectiveness test results of the algorithm are shown in Figure 7.

In order to verify the effectiveness of SDN network traffic prediction model based on speech recognition, this chapter designs several simulation experiments on the MATLAB simulation platform. Experimental results show that the effectiveness of this algorithm can reach 95.67% at the highest, and the MSE convergence is 0.0021 at the lowest. The results show that this model has faster iterative convergence speed, better training effect, and higher prediction accuracy.

## 5. Conclusions

The development of voice technology will further help education and teaching, improve teaching efficiency and teaching effect, and promote the overall development of artificial intelligence technology. Based on the research of related literature, this paper constructs a SDN network traffic prediction model based on speech recognition and applies it to the educational information optimization platform. In this paper, the data acquisition module in the SDN controller is designed, and the SDN network platform is built, which realizes the data acquisition and storage of traffic situation indicators. Aiming at the problems of packet disorder, this paper proposes an IRS mechanism based on the advantages of SDN centralized control and the characteristics of different performance requirements of large and small streams. For large streams, IRS uses greedy routing and multipath routing based on remaining bandwidth to make the traffic evenly distributed in the network, and IRS adds a scheduling strategy based on IP addressing to avoid packet disorder. Simulation results show that the effectiveness of this algorithm can reach 95.67% at the highest, and the MSE convergence is 0.0021 at the lowest. The algorithm can effectively realize the relative priority of each packet queue without reducing the utilization of network resources and can smoothly upgrade and downgrade the priority. On the whole, this model has faster iterative convergence speed, better training effect, and higher prediction accuracy. This research can provide some technical support for the educational information optimization platform. Although this paper has achieved some research results, the research is still shallow, and there are still many places that need to be improved and supplemented. In the future research, we should find a unified solution in different algorithms so that the performance of packet delay and system throughput is better and the utilization of network resources is maximized.

## Figures and Tables

**Table 1:** Parameter setting of the simulation experiment.

Serial number	Parameter	Set up
1	Link bandwidth	10 Mbps
2	Link delay	3ms
3	Sending node	S1, S2
4	Receiving node	D1, D2
5	Minimum threshold	50
6	Maximum threshold	100
7	Maximum packet loss probability	0.2
8	Weight	0.03

**Table 2:** Test results of each index.

Index	IRS	ECMP
Network throughput	1058	957
Average flow completion time	48	53
Remaining bandwidth jitter of Pod link	26	30
Core layer link remaining bandwidth jitter	20	24

## Data Availability

The data used to support the findings of this study are available from the corresponding author upon request.
